# A Fast and Low-Impact Embedded Orientation Correction Algorithm for Hand Gesture Recognition Armbands

**DOI:** 10.3390/s25072188

**Published:** 2025-03-30

**Authors:** Andrea Mongardi, Fabio Rossi, Andrea Prestia, Paolo Motto Ros, Danilo Demarchi

**Affiliations:** Department of Electronics and Telecommunications, Politecnico di Torino, 10129 Turin, Italy; andrea.mongardi@polito.it (A.M.); fabio.rossi@polito.it (F.R.); paolo.mottoros@polito.it (P.M.R.); danilo.demarchi@polito.it (D.D.)

**Keywords:** embedded algorithm, hand gesture recognition, human–machine interface, surface electromyography, wearable armband

## Abstract

Hand gesture recognition is a prominent topic in the recent literature, with surface ElectroMyoGraphy (sEMG) recognized as a key method for wearable Human–Machine Interfaces (HMIs). However, sensor placement still significantly impacts systems performance. This study addresses sensor displacement by introducing a fast and low-impact orientation correction algorithm for sEMG-based HMI armbands. The algorithm includes a calibration phase to estimate armband orientation and real-time data correction, requiring only two distinct hand gestures in terms of sEMG activation. This ensures hardware and database independence and eliminates the need for model retraining, as data correction occurs prior to classification or prediction. The algorithm was implemented in a hand gesture HMI system featuring a custom seven-channel sEMG armband with an Artificial Neural Network (ANN) capable of recognizing nine gestures. Validation demonstrated its effectiveness, achieving 93.36% average prediction accuracy with arbitrary armband wearing orientation. The algorithm also has minimal impact on power consumption and latency, requiring just an additional 500 μW and introducing a latency increase of 408 μs. These results highlight the algorithm’s efficacy, general applicability, and efficiency, presenting it as a promising solution to the electrode-shift issue in sEMG-based HMI applications.

## 1. Introduction

Hand gesture recognition has been a really hot topic in recent years in research on Human–Machine Interfaces (HMIs) [1]. Indeed, many researchers around the world focus on it to ease human life in multiple scenarios [2], ranging from virtual and augmented reality for games or real-life simulations [3] to dedicated medical applications like hand prostheses control, with minimal or no invasiveness to the humans wearing the devices [4].

In all these fields, state-of-the-art works propose plenty of sensors and interfaces to build HMI systems following the user and application requirements [5,6,7]. However, researchers are still struggling to design optimized solutions that could adapt to different people in an easy and friendly way, without necessarily requiring recourse to skilled support. In fact, the majority of both industry and research devices available nowadays need an adaptation period to fine-tune the parameters and train the subject in how to use the medical device itself [8].

Among all the possible sensor configurations, surface ElectroMyoGraphy (sEMG), i.e., the possibility to sense the contractions of skeletal muscles [9], is a natural solution for human-friendly interfaces [10], but it is still affected by the problem mentioned above. Indeed, the people using sEMG-based HMI systems have to generally wear sensors, e.g., wearable armband devices [4,11,12,13], on the forearm to detect the muscular activity underneath and decode this information to understand the corresponding hand gestures. However, the acquired information varies greatly depending on people’s forearm morphology and, considering medical applications, patients’ health conditions [14]. To take into consideration this variability, it is common practice to integrate machine learning routines into the sensors’ logic to automatically generalize data acquired from different subjects while ensuring overall reliability and robustness to outlier phenomena [15,16]. However, any machine learning model is able to perform according to the data space it is based on, and its performance is still susceptible to out-of-the-space data. For the HMI systems based on sEMG, the machine learning training database, resulting from a data acquisition process, is usually built by standardizing the precise location of sensors with respect to the muscles they sense. It follows that the HMI system could not respond as expected in the presence of sensors’ displacement from their reference position, a typical situation when the user wears and doffs the system multiple times [7,17,18,19].

Different strategies to manage the aforementioned issues have been reported in state-of-the-art works, ranging from data augmentation techniques [20,21] to sensor fusion integration [22,23,24] and sensor augmentation approaches [25]. Although all of them report promising results depending on the case studies of reference, their applicability cannot always be guaranteed in all HMI applications, especially for the ones where embedded computation is required. Indeed, data augmentation extends data space to train machine learning models and can result in more complex architectures, which only sometimes fit in resource-reduced hardware like MicroController Units (MCUs). The use of additional sensors and signals, e.g., inertial motion units to assess body kinematics, could require changes in the hardware setup, which could not be achieved in every scenario. Similarly to the previous point, augmenting the sensor number, e.g., using an array of sensors and exploiting the benefits of high-density sEMG, gives the user a better and complete representation of muscular activation but, again, needs complex hardware modifications, which could lead to incompatibility with wearable, everyday use systems. An additional, complementary strategy proposes to include the data corresponding to the electrode shift directly in the training database by carrying out acquisition campaigns with the HMI system voluntarily shifted, in a way to develop machine learning models able to deal with this situation [18,26,27]. On the one hand, following this approach, the resulting solutions perform better in the electrode shifting conditions, but, on the other hand, constructing these databases requires a massive effort in terms of the data campaign.

Indeed, inter-subject diversity is addressed by involving as many people as possible in the data acquisition process, intra-subject variability is considered by repeating the same task several times, and the HMI shift conditions still require additional trials. All this entails a demand for time and human resources that is only sometimes available for research groups, thus limiting the adaptability of some of these solutions.

Considering the scenario described above, in this paper we propose a novel approach for handling the misplacement issue when using hand gesture HMI systems based on forearm wearable sEMG armband devices. In order to extend the adaptability of our approach to other works, we defined and implemented it as an independent packet without any additional requirements in terms of hardware, database extension, and machine learning model modifications. Indeed, our solution consists of the definition of two reference, database-specific, hand gestures to be performed during a calibration phase to understand the device orientation w.r.t. forearm muscles, combined with a real-time correction algorithm to manage the classifier input data before proceeding with the prediction. The calibration process was designed to take place directly after wearing the armband and before its operational use, with the aim of making it quick and easy enough to be applied on any occasion. The orientation correction algorithm was conceived as an operation to be inserted between the data acquisition/feature extraction process and the gesture prediction in a way to avoid any modification to the previous and subsequent stages. Both the computational latency and cost of this operation have been taken into account in order to fulfill the real-time requirement of HMI systems and to allow the algorithm to be implemented in embedded solutions.

After a brief introduction to our custom device [28] in Section 2, the basic principles of our orientation estimation algorithm are reported in Section 3, presenting the preliminary analysis we have performed on multiple databases to define our general and adaptable approach. Then, Section 4 shows how to implement the proposed orientation estimation algorithm in our case study. This consists in the use of our custom-developed sEMG-based armband [28] for the classification of nine hand gestures in a scenario involving 25 subjects while using the device without paying attention to its preferred orientation. Our results and their analysis are reported in Section 5, while a comparison with similar approaches is discussed in Section 6. Lastly, Section 7 summarizes the work and anticipates future developments.

All the in vivo experimental tests we conducted for this work were performed on healthy volunteers, after asking for their informed consent, and strictly following protocol n. 445136, approved by the Comitato Bioetico di Ateneo of the University of Turin [29].

The following list reports the novel contributions and the strength points introduced with this work:

- A new orientation estimation algorithm, consisting of a pre-operational calibration and an online correction phase, has been proposed;
- The algorithm can be adapted to different pre-existing databases without the need to introduce new hardware, conduct additional data acquisition campaigns, or re-train machine learning models;
- All the computations needed to run the algorithm (both calibration and correction) can be embedded into an MCU, making it versatile to be implemented in both software and firmware standalone routine code;
- The proposed calibration process lasts less than 1 min and requires the subject to perform only two simple movements, making it quick and user-friendly;
- We customized and implemented the proposed algorithm for our HMI armband [28], testing it on 25 subjects and achieving a global classification accuracy of 93.36% with the device worn in arbitrary orientation, and also obtaining a minimal additional latency of 0.408 ms and a power consumption increment of only 500 μW when introducing the algorithm.

## 2. Previous Work on Our sEMG-Based Armband

This section aims to introduce the relevant aspects of our past publication on the custom sEMG-based armband for gesture recognition [28], where we presented all the design, development, and validation phases for the realization of the device, to better contextualize what will be reported in the following sections.

**Figure 1:**
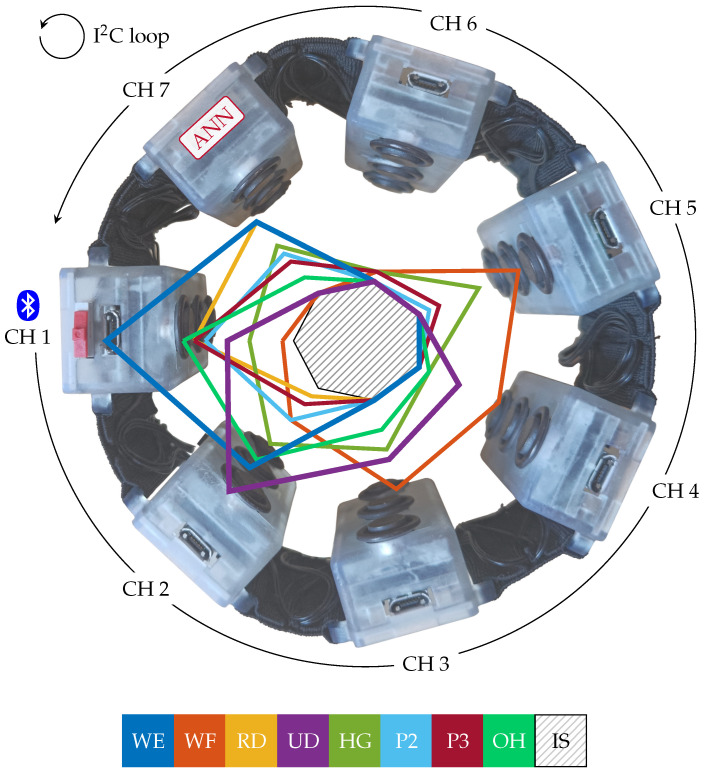
Armband device structure: the representation shows the 7 channels (CH 1–CH 7) and the wired (I^2^C) and wireless (BLE) communication protocols implemented. The overlapped profiles represent the ATC values distribution for the nine hand gestures recognized by the system: wrist extension (WE), wrist flexion (WF), radial deviation (RD), ulnar deviation (UD), hand grasp (HG), thumb–index finger pinch (P2), thumb–middle finger pinch (P3), open hand (OH), and idle state (IS). The ATC profiles are based on the data acquired on 20 subjects in our previous work [28].

As represented in Figure 1, the armband is composed of seven equally spaced modules (CH 1–CH 7), connected to one another by an I^2^C closed-loop daisy-chain configuration, for the sensing, processing, and transmission of the muscular information. Each module contains the same hardware, consisting of the sockets for clipping the Ag/AgCl dry electrodes to the armband’s case in a sensing–reference–sensing configuration, with an inter-electrode distance of 1.8 cm and a single custom Printed Circuit Board (PCB) [30] featuring all the electronics components. These include the Analog Front-End (AFE) for the sEMG signal, power management circuitry, and the MCU with its Bluetooth Low-Energy (BLE) transceiver. The AFE provides a flexible sEMG amplification in the 250–4000 V/V range, user-selectable at steps of 250 V/V, and a band-pass filter for the 30–400 Hz frequencies, in addition to input and output shields components [30]. The MCU, i.e., the Apollo3 Blue System-on-Chip (SoC) [31], is configured via firmware to enable data acquisition, wired and wireless transmission, or gesture classification tasks depending on the module roles. Communication between the armband and external devices is via the BLE communication protocol, operated solely by CH 1. All the electronic components are compliant with a standard 3.7–4.2 V battery power source, and the modules drop out the operating voltage to 1.8 V to limit power consumption. A single 175 mAh LiPo battery [32] powers the entire device.

All the seven channels implement the event-driven Average Threshold Crossing (ATC) technique [30,33,34], and the ATC feature thus obtained is the only indicator of muscle activity acquired by our channels, which do not even sample sEMG. The ATC technique is implemented entirely in hardware and consists of counting the number of times the analog sEMG signal crosses a positive threshold within time windows. In particular, a voltage threshold comparator at the end of the AFE generates an event every time the analog sEMG crosses the threshold: the result of this stage is the quasi-digital threshold crossing (TC) signal, i.e., a digital signal where the rising and falling edges are at the instants of threshold crossing. The TC signal is then used as the clock source for a timer of the MCU operating in counter mode. Channels whose sole purpose is the acquisition of muscle activity can then operate in sleep mode to save energy and activate at the end of each ATC acquisition window, which in our case is 130 ms, to forward the number of events counted by the timer (i.e., the ATC feature). A dedicated threshold calibration algorithm has been developed to automatically find a suitable threshold for the TC generation, taking into consideration AFE settings and environmental noise conditions [30]. Considering the above strategy, our armband does not directly sample and process sEMG data but computes the ATC feature, which drastically reduces data payload, processing time, and related power consumption w.r.t. standard sampling and transmission methodologies [28,30].

As discussed in [28], we implemented an ATC-based Artificial Neural Network (ANN) directly on the armband, in CH 7, to recognize nine hand gestures. We chose ANNs because they are computationally lighter and faster than other classification algorithms, especially when performing on embedded devices, and we demonstrated how the combination of the ATC method and ANN resulted in a very slim and simple network. The best ANN architecture we found [28] was composed of four layers, i.e., the input layer composed of 7 neurons (the armband channels), followed by two hidden layers of 50 neurons each, and ending with the output layers consisting of 9 neurons representing the nine hand gestures to be classified. Figure 1 reports the median ATC activation profiles distribution (20 subjects) across channels for the following target gestures: wrist extension (WE), wrist flexion (WF), radial deviation (RD), ulnar deviation (UD), hand grasp (HG), thumb–index finger pinch (P2), thumb–middle finger pinch (P3), open hand (OH), and idle state (IS).

We validated our armband by carrying out an online test campaign on six subjects and achieved an overall average classification accuracy of 91.93% on the nine hand gestures above, supported by a short prediction latency of 1.342 ms and an average current absorption of only 2.920 mA. However, during our previous tests, proper operation of the armband required adherence to a reference-wearing orientation, particularly with the device rotated so that the CH 1 module was placed over the extensor digitorum muscle. Failure to meet this requirement would result in a drastic performance decrease (quantitative information on this is provided in the next section). We therefore found it necessary to develop the orientation correction algorithm presented in this paper, so as to allow arbitrary wearing of the device. It is worth pointing out that, although our armband relies on the ATC feature, this is not a requirement for using the proposed algorithm, which is designed to work with any indicator of muscle activity, such as standard sEMG features like Root Mean Square (RMS).

## 3. Algorithm Definition

**Figure 2:**
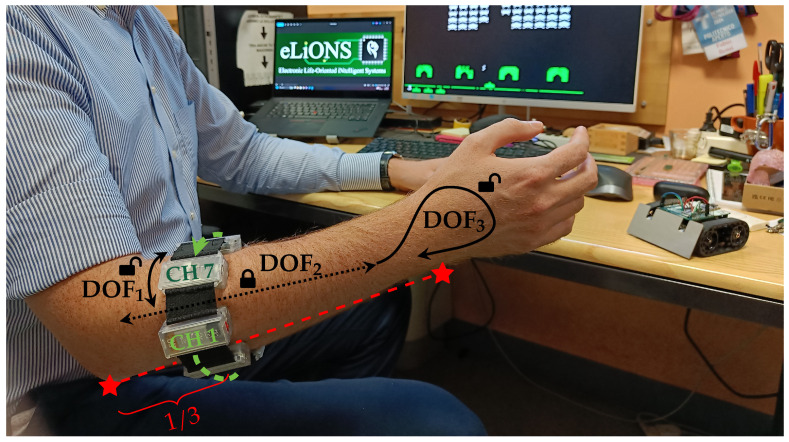
A general user wearing our custom sEMG armband. Considering its three degrees of freedom (i.e., DOF_1_—rotation around the forearm, DOF_2_—lengthwise position, and DOF_3_—reversed insertion), the proposed embedded calibration algorithm is able to compute both DOF_1_ and DOF_3_ information, making the wearing of the device easier than searching for the exact reference position. The forward and backward shift of the armband along the forearm (DOF_2_) is instead fixed one-third along the length from the elbow to the wrist in order to sense the muscular signals where muscle bodies are more accessible.

An armband for hand gesture recognition has three degrees of freedom (DOF), as represented in Figure 2: it can be rotated around the forearm (DOF_1_), moved backward and forward (DOF_2_), and inserted back to front (DOF_3_).

However, moving the armband too much along the forearm (DOF_2_) stops making sense if the device moves away from the muscle-sensing zone. Indeed, forearm muscle bodies are primarily located in the proximal region of the elbow, and their extension toward the wrist mainly consists of the tendon extremities. That is the reason why different producers [4,12,13] or researchers [26,35,36,37] study and develop armbands that work on and fit to the proximal forearm, although some preliminary works are starting to evaluate how custom devices perform at the wrist site [11,38]. Therefore, in order to follow the standard approach, we decided to proceed with our evaluation by focusing only on the proximal region of the forearm, thus considering databases [39,40] and studies [16,41] that have been developed by locating the armband sensors along approximately one-third of the segment identified between the elbow and the wrist, starting from the elbow. Nevertheless, since in this region the muscle fibers evolve quite parallel to the forearm axis, the limited variation in sensors placement along DOF_2_ does not significantly alter the detected signals. For this reason, we decided not to correct the lengthwise position, leaving its handling to the machine learning model processing the muscular activation.

**Figure 3:**
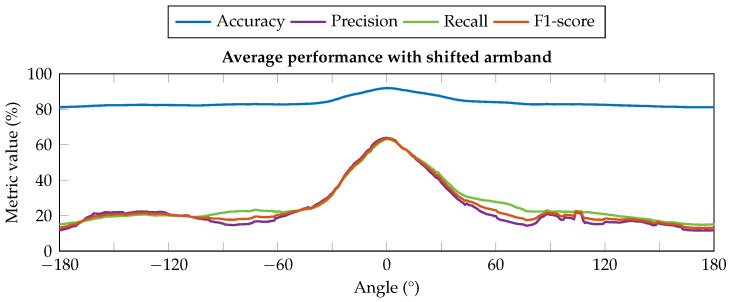
Classifier performance with different artificial shift conditions of the input data. All the metrics suffer a relevant decrease in their values when the armband is rotated by about ±30° from the reference position (0°), significantly impacting the reliability of the classification outcomes.

On the contrary, the angular shift of the armband around the forearm (DOF_1_) leads the acquisition channels to monitor muscles different from those expected from the reference position, i.e., with CH 1 above the extensor digitorum muscle. To demonstrate how the effect of DOF_1_ shift from its reference position could affect the system performance, we carried out the following offline analysis to point out the problem when using our armband. The dataset we collected on six subjects in the testing phase of our previous work [28] was rearranged on MATLAB^®^ version R2019a to obtain multiple orientations from each sample. The shifted data were then given as input to the same ANN already used in the testing phase, aiming to classify the nine hand gestures introduced in the previous section, obtaining the classification performance reported in Figure 3. The performance at the reference position (shift angle equal to 0°) corresponds to that obtained in the previous work, with an average accuracy equal to 91.93% and the other metrics equal to about 63%. Looking at the performance profile trends, it is possible to observe how all the metrics rapidly decrease by about ±30° from the reference position and then flatten. In particular, the average accuracy decreased by about 10%, resulting in a value of 83.8%, and the precision, recall, and F1-score decreased by more than 40% w.r.t. the optimal value. This outcome we obtained, similar to other results in the same field [8,16,41,42,43], confirms the need to estimate and correct the angular shift when the device is worn differently from the reference position.

Similarly, the reversal condition (DOF_3_) overturns, symmetrically or not depending on the coexistence of the DOF_1_ shift, the acquired muscle information w.r.t. those expected by the classification model, resulting in unreliable misclassification results. Although the probability of reversal is lower than other shifts, and may be limited by users’ awareness, we still wanted to address this point in order to give users the opportunity to wear the armband as naturally as possible.

According to the above considerations, we defined an estimation and correction algorithm for both DOF_1_ and DOF_3_, which takes place in two phases: first, the user can casually wear the armband, just paying attention to respect the approximate lengthwise position, and then s/he has to perform a calibration procedure executing one or more gestures for a short period (e.g., tens of seconds) before starting standard operations. Thus, once both shift (DOF_1_) and reversed (DOF_3_) conditions have been assessed, the device is aware of its relative orientation, the sensors’ data can be corrected, and then the hand gesture classification process can proceed normally without any additional modification.

### 3.1. Calibration Algorithm Formulation

**Figure 4:**
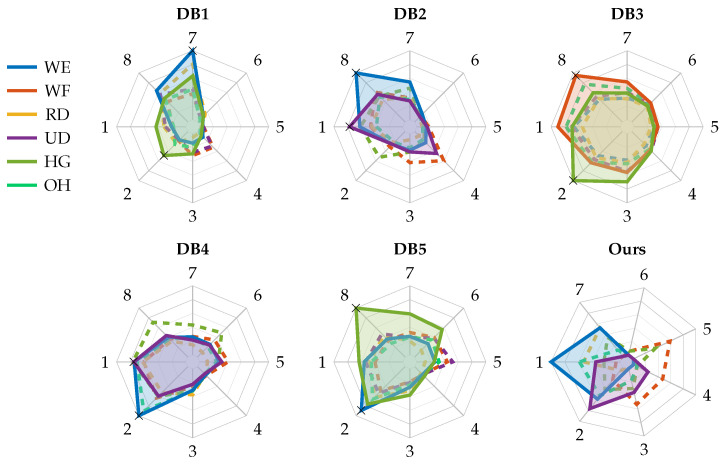
Analysis of activation profiles of Ninapro publicly available databases [39,40]. Attributes of each database are given in Table 1. For each candidate gesture for the calibration routine, the figure shows the distribution of RMS profiles across the sensing channels. The plotted profiles in each radar chart were normalized with respect to their maximum value. The highlighted gestures are those identified as suitable for the calibration routine, while the black cross markers are the points to be used to correct the reversal wearing. The last radar chart is the distribution of ATC activation profiles of our database [28].

As introduced above, our proposed solution aims to correct the angular shift and reversal condition of sEMG-based armbands for hand gesture recognition. To define a correction algorithm suitable for our case study and generalizable to other devices, we performed an analysis leveraging the first five Ninapro databases [39,40], namely DB1, DB2, DB3, DB4, and DB5. The analysis thus involved data acquired with OttoBock [44], Delsys [45], and Cometa [46] double-differential sEMG electrodes and with the Myo armband [4]. We chose to include the Myo armband in this analysis because of its widespread use [47,48,49,50,51], but we acknowledge that the Myo armband has a poor sampling frequency of 200 Hz and is therefore susceptible to aliasing, as the sEMG signal has the greatest frequency contribution in the 50–150 Hz range [52]. A previous study [53] also showed how a sampling frequency of 200 Hz, compared with 1000 Hz, results in a drastic reduction in classification performance. For the purpose of the analysis, only sensors arranged around the forearm at the height of the radio humeral joint, following a circular configuration, were considered, thus excluding those placed on the biceps brachii and triceps brachii, or on the specific spots of the flexor and extensor digitorum superficialis, and considering only one armband in cases where two were used. Therefore, the first eight channels of each database were involved in this investigation. Among all the gestures included in the Ninapro databases [39], our analysis considered only the most meaningful gestures for a calibration routine, i.e., open hand (exercise B, gesture 5), hand grasp (exercise B, gesture 6), wrist flexion (exercise B, gesture 13), wrist extension (exercise B, gesture 14), radial deviation (exercise B, gesture 15), and ulnar deviation (exercise B, gesture 16), and thus excluding selective finger movements. To expand our analysis beyond healthy subjects, we also included data from the subjects with amputations included in DB3, of whom we considered only people with right-hand amputations for consistency with the other databases where data acquisition was performed on the right arm. The attributes of each database involved in this investigation are summarized in Table 1. For each Ninapro database, the RMS profiles of the provided sEMG signals were computed as the indicator of muscle activity, with the only exception of DB1 where the provided signals are already RMS profiles. The RMS was computed over sliding windows of 200 ms, with a moving step equal to 10% of the window length, i.e., 20 ms. Then, the RMS signals were segmented according to the provided restimulus labels, and the median of the RMS values was computed for each movement, for each channel, analogous to what was done with the ATC values in our database [28] to identify the ATC profiles distribution across the channels shown in Figure 1. The results of this process are reported in Figure 4, showing the activation profiles of each channel for each Ninapro database under analysis and for our ATC database. Since the signal amplitude ranges of each database differ from each other either because of the use of different acquisition systems or due to the presence of people with amputations, the values of each radar chart in Figure 4 was normalized with respect to the maximum value plotted in each. We chose to use RMS and not to software-extract ATC from the databases under analysis because, as mentioned, ATC is not a requirement to assess the armband orientation; we use it here because it is the feature used by our device, but its software extraction does not necessarily reflect what can be obtained in hardware (the sampling frequency negatively affects the ATC quality). The only requirement for choosing the feature to be used for orientation estimation is its high correlation with muscle activity level.

Looking at the obtained charts, we identified two necessary and sufficient conditions to be satisfied during the calibration routine to handle the angular shift and reversal corrections, respectively:

(A) Presence of a movement with an arrow-shaped activation profile, i.e., with a peak of activation in one of the sensing channels and reduced activity moving away from it.
(B) Presence of a movement, distinct from that in condition A, featuring at least one activation peak located in a position that is neither the same as in condition A nor its opposite.

**Figure 5:**
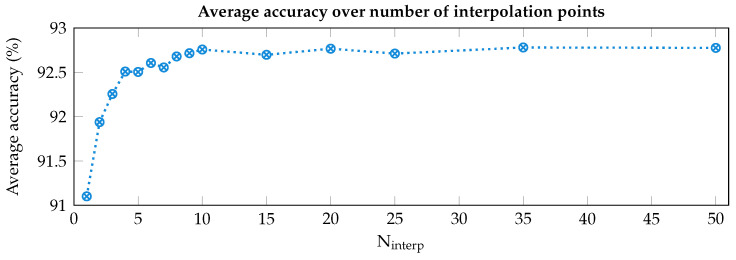
Accuracy of the system for different resolutions (i.e., number of interpolation points, N_interp_, between adjacent channels) of the orientation estimation algorithm.

The movement identified to meet condition A is wrist extension (WE) in most cases (see Figure 4), with the only exception in the dataset of people with amputations (i.e., DB3), where the movement identified is hand grasp (HG). To test whether leveraging the activations occurring during WE can identify and correct the angular shift, a preliminary acquisition was performed using our armband on a single subject and the following offline analysis was performed. We selected the median WE profile obtained after the acquisition campaign of our previous work [28] as reference in order to compute the angular shift between it and the WE profile obtained during the calibration phase. To increase the shift resolution and not be limited to the poor inter-channel distance of 51.4° (i.e., 360°/seven channels), the profiles were interpolated with a cubic spline operator. This method was selected considering the continuous variability of the muscular activations, thus trying to minimize the error due to information loss when the active muscle(s) lie in the middle of two sensing channels. Indeed, since the forearm is composed of very dense muscular fibers, involving a linear interpolation, or other similar computationally light methods, would have removed important muscle contributions from the derived model. Both acquired and reference profiles were interpolated multiple times, varying the number of interpolation points (N_interp_) between two adjacent channels from 1 to 50, thereby increasing angular resolution from 51.4° to 1.03°, respectively. The angular shift was then estimated by cross-correlating the two interpolated profiles. Following this step, the subsequently acquired profiles were first interpolated, then shifted based on the estimation, and finally resampled to obtain seven values, each at the position of an armband channel. Each set of data thus obtained was given as input to an ANN identical to the one embedded in our armband to simulate the possible outcomes in terms of prediction accuracy. The resulting curve (in Figure 5) flattens for values of N_interp_ greater than 10 (i.e., a resolution of 5.14°), and we therefore chose this value for the interpolation step.

In contrast to condition A, where the choice to include wrist extension in the calibration routine is straightforward given the shape of its activation profiles, for the fulfillment of condition B the choice is less obvious, and heterogeneity among the analyzed databases leads to different choices being made, mainly in favor of ulnar deviation (UD) or hand grasp (HG). Indeed, these movements result in the most prominent activation peaks beyond wrist extension while fulfilling condition B. Then, to identify if the armband is worn reversal or not, the D_ch_ parameter is defined, equal to the distance (in terms of channels) separating the peaks of the two gestures selected for the calibration routine. Indeed, knowing the theoretical distance of their activation peaks from the reference database, it is sufficient to rotate the profile of the second gesture (i.e., the one selected to satisfy condition B) forward and backward by a number of channels equal to D_ch_, and calculate the Mean Absolute Error (MAE) between the rotated profile and the profile of the first gesture (i.e., the one selected to satisfy condition A). The reversal wearing is then identified if the relationship between MAE forward (MAE_fw_) and MAE backward (MAE_bw_), e.g., MAE_bw_ < MAE_fw_, during the calibration routine does not match the relationship of the theoretical data from the reference database. Essentially, for each database we can define flag_bw_ equal to 1 if MAE_bw_ < MAE_fw_, or 0 otherwise. Then, the identification of reversal wearing is achieved according to (1):

(1) $$\begin{array}{cc} \mathrm{Reversal}=\; & \left(\left({\mathrm{MAE}}_{\mathrm{bw}}>{\mathrm{MAE}}_{\mathrm{fw}}\right)\wedge \left({\mathrm{flag}}_{\mathrm{bw}}=1\right)\right)\\ \; & \vee \;\left(\left({\mathrm{MAE}}_{\mathrm{bw}}<{\mathrm{MAE}}_{\mathrm{fw}}\right)\wedge \left({\mathrm{flag}}_{\mathrm{bw}}=0\right)\right) \end{array}$$

For each database analyzed in this work, the activation peaks of interest of the two gestures identified for the calibration routine are marked with black crosses in Figure 4, while Table 2 reports the corresponding D_ch_ values, and the resulting flag_bw_ obtained subsequent to the calculation of MAE_bw_ and MAE_fw_ among the reference activation profiles.

### 3.2. General Application Example

As a clarifying example that summarizes what was defined above, let us suppose a scenario where a new armband made of eight Delsys channels arranged around the forearm has been developed for an HMI, and a new database to train its hand gesture classification algorithm cannot be recorded (e.g., lack of available subjects). The user could then take advantage of an ANN previously trained on DB2, but without having to worry about complying with the exact electrode placement observed for the creation of DB2 while wearing the new armband, as long as it was worn at the height of the humeral radio joint (i.e., the only constraint on DOF_2_). By performing a calibration routine following the armband donning, it would be possible to align the new data with that of DB2, thus not requiring the re-training of the ANN already available. Considering these assumptions, for this scenario it would be convenient to choose WE and UD as calibration gestures (see Figure 4) for satisfying conditions A and B, respectively. Performing these gestures during calibration would allow the identification of reversal wearing by computing if MAE_bw_ > MAE_fw_, and subsequently the estimation of angular shift by computing the cross-correlation between the theoretical WE profile from DB2 and the interpolated WE profile just recorded (mirrored, if reversal was identified). Knowing these estimates, all the features required for the gesture recognition algorithm would be aligned in real-time while using the new armband. More details on the implementation of the proposed approach are provided in the next section.

## 4. Algorithm Implementation and Validation

To validate the functionality of the proposed approach, we implemented the orientation algorithm on our HMI system. To summarize, it consists of a wearable seven-channel armband, with an embedded ATC-based ANN able to recognize nine hand gestures, and dedicated software for guiding the user during operation and saving data. Other essential details are reported in Section 2, while for a more complete discussion please refer to our previous publication [28]. Considering the ATC activation profiles distribution of Figure 4, and the data reported in the bottom row of Table 2, we selected the wrist extension (WE) and ulnar deviation (UD) gestures for satisfying conditions A and B, and we chose D_ch_ equal to 1 for the MAE computation.

Next, Section 4.1 and Section 4.2 demonstrate how to implement both the calibration and correction phases into an existing environment, here specific for our system but generalizable to any other solution respecting the requirement discussed in Section 3.1. Lastly, our experimental setup for testing the algorithm performance is reported in Section 4.3.

### 4.1. Calibration Algorithm Implementation

**Figure 6:**
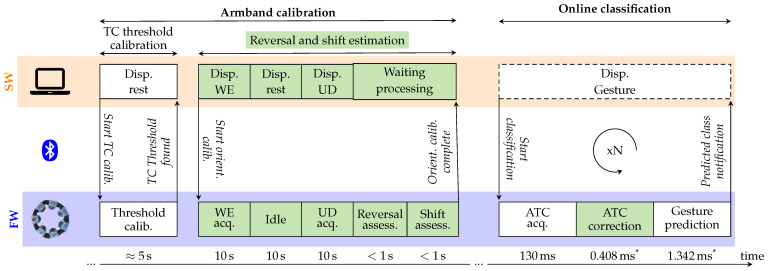
Software (SW) and firmware (FW) interconnected operations for the calibration and online classification working phases. The green blocks represent the new steps for estimating the orientation of the device and for correcting the sensed values before performing the classification. All the algorithm computations are embedded on the armband modules’ MCUs while the SW package runs the graphical user interface (GUI), helping the user to follow the indications for a straightforward experience. The bottom timeline also reports the developer-defined calibration protocol timings and the (*) system latency measured during experimental validation.

The calibration algorithm was integrated into the existing software (SW) and firmware (FW) modules [28]. Considering the operational flow reported in Figure 6, the armband orientation estimation, i.e., reversal and shift computing, takes place directly after the TC threshold calibration, thus making the overall calibration process more complete. It consists of the real-time acquisition of the ATC data while the user is performing WE and UD movements and in the data processing to extract the orientation information. After the power on and BLE connection of the armband with the central unit (e.g., a laptop), and once the TC threshold has been established, the user has to maintain WE and UD movements for 10 s each, separated by resting phase of 10 s also.

On the software side, we updated all the processes and the graphical user interface (GUI) to guide the user in the sequence of gestures and rest phase needed to complete the calibration. The images of the gestures to be performed, a timer representing the activation/rest intervals, and additional information about the status of the calibration process are displayed on a dedicated software window.

All the computations needed to obtain the orientation of the armband were embedded in the firmware of the device and split into three sequential stages for the ATC profile acquisition, and the determination of reversal and shift conditions, respectively. All the stages were assigned to separated tasks, following the FreeRTOS logic [54], in order to functionally divide the workload and follow the timing of the entire process, which is dictated by the software. As in [28], the new calculations were implemented employing the CMSIS DSP package [55] to optimize the hardware execution of the needed floating-point mathematical operations.

The first stage corresponds to the ATC data acquisition of both the WE and UD profiles. Since the developed armband’s Application Programming Interface (API) [28] already defines all the modules’ actions to perform this task, we just needed to implement the timing schedule of the protocol and to label the acquired data opportunely. The WE and UD profiles used for the following processing stages are obtained by applying the median operator along the channels’ dimension over the central 5 s of gesture active duration to limit borderline effects: considering the standard ATC window of 130 ms and the 5 s activity clip, the acquired 38 × 7 ATC activation matrix (i.e., N_ATCwindows_ × N_channels_) is reduced to the 1 × 7 median ATC array.

**Figure 7:**
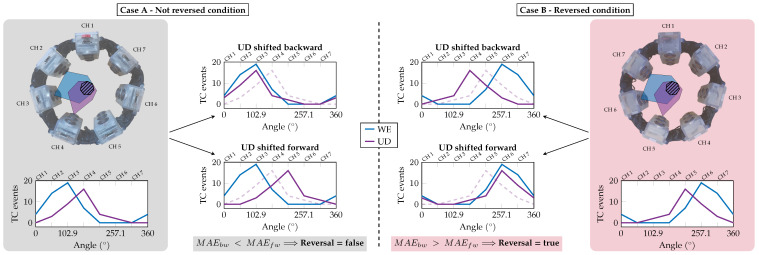
Reversal assessment: the reversal condition is determined depending on the position of the UD peak w.r.t. the WE peak. In particular, the Mean Absolute Error (MAE) has been computed by shifting the UD profile forward and backward one channel and comparing it with the WE profile. If the error in the backward position (MAE_bw_) is lower than the forward one (MAE_fw_), the armband is worn in the correct non-reversed condition (case A), otherwise a reversal condition is detected (case B).

The second stage, the reversal assessment, starts directly at the end of the previous one and, by processing the ATC data acquired for both WE and UD movements, verifies their relative position. As graphically represented in Figure 7, the UD profile is shifted one channel forward and backward, and the respective MAE (i.e., MAE_fw_ and MAE_bw_) with WE are computed. According to (1) in the case of flag_bw_ equal to 1, since with the standard orientation the UD peak would be located one channel after the WE peak (Figure 7, case A), MAE_bw_ should be lower than MAE_fw_ when the armband is worn in the original direction. If MAE_bw_ is greater than MAE_fw_ (Figure 7, case B), a reversal condition is detected, the corresponding boolean variable is set (Reversal = true), and all the ATC profiles henceforth acquired will have their values swapped to restore the correct shape w.r.t. the reference profile.

**Figure 8:**
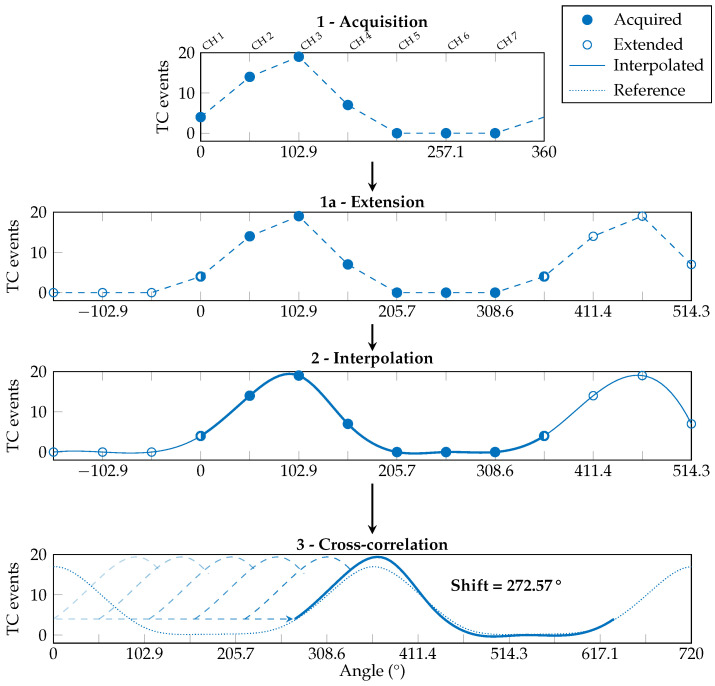
Shift assessment: the estimation of the armband shift only requires the processing of the WE profile. The acquired ATC values (1) are extended with 3 more points at its extremities (1a) to be interpolated with a cubic spline function (2). Then they are cross-correlated with the reference WE profile (3) to find the angular shift of the armband.

The third stage, named shift assessment, runs immediately after computing the reversal condition and performs the evaluation of the angular shift involving only the WE profile. Following the steps reported in Figure 8, before proceeding with the cubic spline interpolation, the acquired median WE profile (step 1) is extended by replicating three values before and after its original domain (step 1a) to ensure that the slopes at the interpolated profile tails are correct. After the interpolation is performed (step 2), only the values between 0° and 360° (i.e., number of channels × N_interp_ = 70 points) are considered.

Then, a cross-correlation is made between the obtained waveform and the reference WE profile, doubled in length (step 3). The angular offset necessary to obtain the maximum value of the cross-correlation corresponds to the angular shift of the armband w.r.t. the reference position. The found shift value is saved into a dedicated variable (e.g., Shift = 272.57°). Once both the reversal condition and shift value have been determined, the device stores the information for when the online classification task will be requested.

All the above considered, and looking at the timeline reported in Figure 6, the entire armband calibration process lasts less than 1 min. Excluding the fixed 30 s orientation calibration user protocol for the acquisition of the WE and UD profiles and the minimal contribution for computing reversal and shift conditions, in this work, we also enhanced the TC threshold searching procedure, making it working simultaneously on all the modules, thus allowing the device to complete the threshold calibration in approximately 5 s (w.r.t. the 30 s of previous sequential implementation [28]).

### 4.2. Orientation Correction During Online Classification

Still considering the flow diagram of Figure 6, now looking at the online classification phase, we can discuss how the orientation calibration takes effect when performing the real-time class prediction.

Our latest implementation [28] worked by acquiring the ATC data from all the seven channels every 130 ms (i.e., the ATC window) and directly feeding the ANN in cascade. As we discussed in Section 2 and Section 3, the ANN has been trained with the dataset acquired wearing our armband in the reference position; therefore, the network fails to classify correctly when the armband orientation changes.

**Figure 9:**
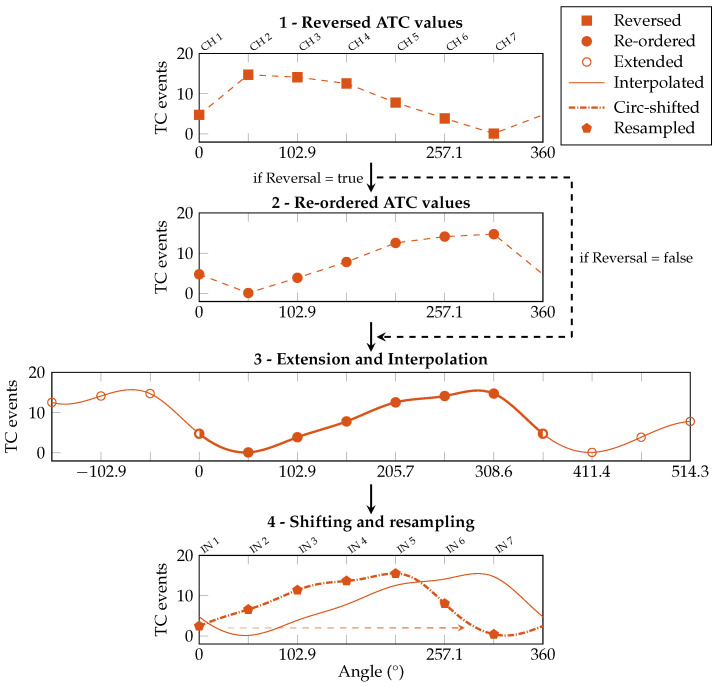
ATC signal correction during real-time operations. First, if Reversal = true, values are symmetrically swapped w.r.t. CH 1 (i.e., CH 2 with CH 7, etc.). Then, the signal undergoes steps 1a and 2 of the shift assessment process. Lastly, the signal is shifted the saved amount of degrees, and resampled with the original channel distance to obtain the seven values (IN 1–IN 7) needed as input by the ANN (4).

In our new implementation, we added the firmware ATC correction between the ATC acquisition and the gesture prediction. This step merges the reversal and shift information obtained during the orientation calibration and modifies the ATC array accordingly. An example of this process is reported in Figure 9, here focused on a different gesture profile w.r.t. the calibration procedure to show the algorithm adaptability, and it works as follows. First, if the reversal condition is detected, the ATC values are symmetrically swapped w.r.t. CH 1 (i.e., CH 2 with CH 7, CH 3 with CH 6, and CH 4 with CH 5). Then, the ATC profile is processed following steps 1 to 2 of the shift assessment routine (here, 2 and 3). Lastly, the obtained interpolated chunk is shifted depending on the calibration value and resampled to obtain the seven ATC values (i.e., IN 1–IN 7) needed as input by the ANN (step 4).

Therefore, the only modification to the online classification process consists of the ATC data rearrangement w.r.t. the found orientation, as described above, while the following prediction process is maintained unaltered, ANN structure and parameters included, as reported in [28]: every 130 ms, the re-oriented ATC data are given as input to the ANN, which outputs the recognized gesture class and communicates it to the master board. Once received, the predicted class is wireless transmitted at the first BLE connection event.

### 4.3. Experimental Validation

To verify the correct behavior of the implemented algorithm, we performed a new experimental validation involving 25 healthy volunteers, including 14 females and 11 males, aged between 23 and 32 years. No exclusion criteria were applied, with one only exception inherent to the armband wearability: due to the physical structure of the armband, its minimal fitting circular (elliptic) diameter is approximately 7 cm and can be extended about + 13% from nominal value thanks to the inter-module elastic bands. The experimental setup involved the subject sitting in front of a monitor, where only the indications of the gestures to be performed were displayed.

**Figure 10:**
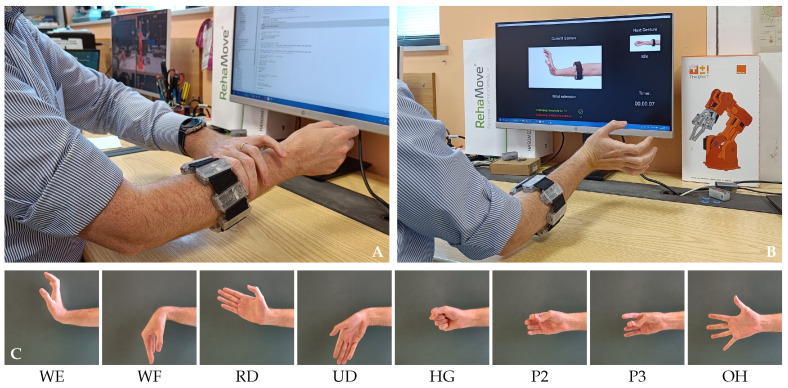
Validation protocol: (**A**) the armband is worn on the right forearm with arbitrary orientation; (**B**) calibration gestures are performed to determine reversal and shift conditions; (**C**) standard gestures are executed to verify the system performances. The testing protocol maintains the same organization of our previous study [28].

After a first explanatory phase, during which the subjects became familiarized with the hand gestures to be executed and the device itself, the participants were asked to wear the armband without paying attention to its orientation (Figure 10A), thus trying to cover the most number of configurations possible. Then, the calibration phase took place, with the gestures and the timing of their execution being provided on the graphical user interface of the subject side (Figure 10B). Once the armband was calibrated and the correct association between the sensed ATC information and the values needed by the ANN was established, the volunteers performed a standard test session involving all the eight active gestures (Figure 10C), as defined in [28]. The test session includes two sequential repetitions of the same gesture to be maintained for 10 s, always separated by a rest interval of 10 s, plus an additional 10 s relaxation before changing the executed gesture. The whole testing session was repeated twice, with the armband being removed and worn again after an inter-session pausing period of 5 min.

## 5. Results and Discussion

After the experimental phase was concluded, we analyzed the system performance to evaluate whether the orientation correction algorithm was beneficial in solving the electrode misplacement issue, and its overall impact on the system.

**Figure 11:**
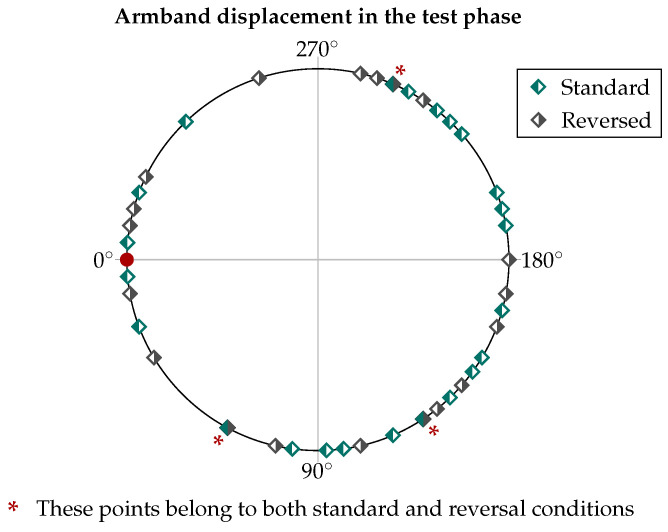
Representation of all the different orientations casually resulting from the test phase, including 23 in the standard wearing condition and 19 in the reverse wearing condition.

**Figure 12:**
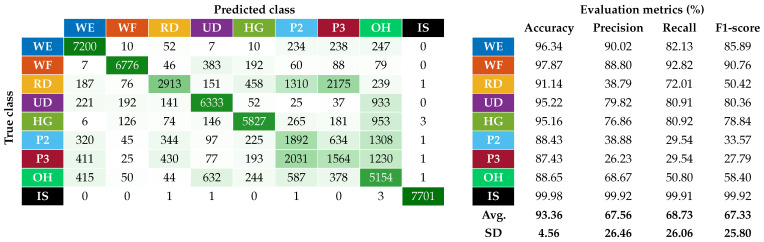
Classification performance of the system after the implementation in real time of the automatic orientation correction algorithm.

The first step was to examine how the classifier worked after the correction of the ATC input data. The test phase included a total of 50 trials (i.e., 25 people times two session repetitions), covering 42 different combinations of shift and reversal conditions, with the distribution depicted in Figure 11. The resulting confusion matrix for the nine classified hand gestures is reported on the left of Figure 12, while the table on the right shows the related metrics with their averages and standard deviations (SDs). In particular, we calculated the accuracy, prediction, recall, and F1-score for each class (c) as defined below:

(2) $${\mathrm{Accuracy}}_{c}=\frac{{\mathrm{TP}}_{c}+{\mathrm{TN}}_{c}}{{\mathrm{TP}}_{c}+{\mathrm{TN}}_{c}+{\mathrm{FP}}_{c}+{\mathrm{FN}}_{c}}$$

(3) $${\mathrm{Precision}}_{c}=\frac{{\mathrm{TP}}_{c}}{{\mathrm{TP}}_{c}+{\mathrm{FP}}_{c}}$$

(4) $${\mathrm{Recall}}_{c}=\frac{{\mathrm{TP}}_{c}}{{\mathrm{TP}}_{c}+{\mathrm{FN}}_{c}}$$

(5) $${F1-\mathrm{score}}_{c}=\frac{2\;\cdot \;{\mathrm{Precision}}_{c}\;\cdot \;{\mathrm{Recall}}_{c}}{{\mathrm{Precision}}_{c}+{\mathrm{Recall}}_{c}}$$

where the number of true positives (TP), true negatives (TN), false positives (FP), and false negatives (FN) are determined for each class (c) using a one-vs-all approach.

The average accuracy and F1-scores achieved were 93.36% and 67.33%, respectively. Compared with what was reported in Figure 3, where for angular offsets greater than 30° the average accuracy and F1-scores were about 83% and 20%, the new results achieved using the proposed correction algorithm allowed the performance to be slightly higher than those obtained complying with the reference position in our previous work [28] (i.e., average accuracy and F1-scores equal to 91.93% and 62.53%). This slight performance increase w.r.t. our previous work revealed how the proposed algorithm, with a resolution of 5.14°, is also able to take into account minimal variations of the armband placement from the reference position, which in our previous work were left to the users’ ability to properly wear the device.

However, the relative performance of the single gestures remained similar to the ones obtained in [28]. In particular, the best performing gestures are still WE and WF, while the worst performing ones remain the finger pinches (i.e., P2 and P3). The two pinch classes are indeed those with the lowest recall values, indicating that our ANN cannot recognize most of their instances. RD on the other hand, although showing higher recall values than P2 and P3, has similarly low precision due to the high presence of false positives, mainly caused by its misclassification with the pinch classes, as can be seen in the confusion matrix.

**Figure 13:**
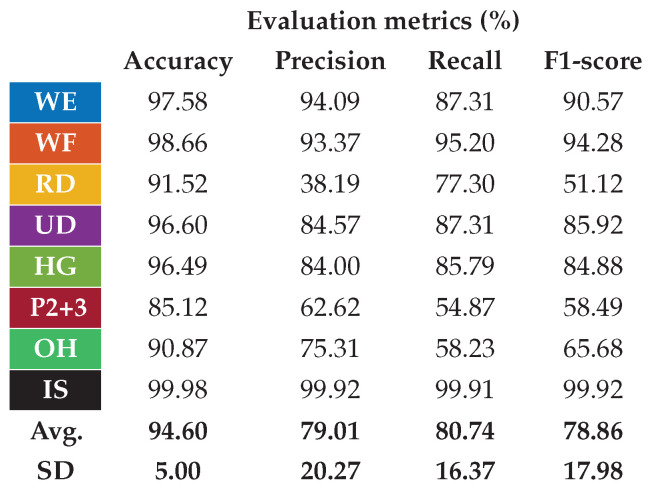
Classification performance improvements if the two pinch classes are merged and a moving window is used on the output class.

Nevertheless, the overall performance can be improved with few changes. The first is the merging of pinch classes P2 and P3, analogous to what was done in [28]. The second involves using short moving windows to strengthen the outgoing class, which is assigned only if the last three predicted classes agree. The results of these two approaches combined are shown in Figure 13, where the average precision, recall, and F1-score are increased by about 17% compared with those reported in Figure 12, with also a decrease in their standard deviation. The use of these approaches in a practical application context was demonstrated in [56].

Latency was also measured during the online prediction phase to verify compliance with the real-time constraints. The measure was performed using a counter timer of the microcontroller, started immediately before the orientation estimation routine, and stopped once the performed gesture was determined. The base latency we obtained in [28] was 1.342 ms, and it took into account the prediction itself only. The obtained measure from this new system implementation was 1.750 ms, which means that the orientation correction itself, performed immediately before the gesture prediction, takes only 0.408 ms. Even adding the 130 ms of the ATC time window and the 50 ms of the BLE connection interval [28] to conservatively estimate the overall system latency, the resulting value, equal to 181.75 ms, is still well under the 300 ms typically considered for real-time control [57].

Lastly, the current absorption of the system was measured during the working phases of the armband to assess the power demand of the proposed calibration routine in real-time operations. The measurements were performed using the DMM7510 7.5 digit graphical sampling multimeter [58], acquiring 20 s of current absorption data during the active prediction process, without and with the orientation correction enabled. The respective mean values of the two modalities were 2.836 mA and 2.895 mA. These measurements confirmed the low power consumption of the armband (i.e., about 12.16 mW), even with this new embedded calibration, which only requires additional 500 μW to the baseline consumption. Thus, the system is still suitable for up to 60 h of continuous wearing [28] with the equipped 175 mAh LiPo battery [32].

## 6. Comparison with SoA Works

In recent research on sEMG-based hand gesture recognition, several studies aimed to improve the robustness of commercial and custom armbands with respect to shifts in their wearing. Four noteworthy works in this field are [16,26,41,43], whose main comparative details are summarized in Table 3.

The strategy proposed in [16] and tested on 10 healthy subjects with the Myo armband allowed the achievement of a significant accuracy improvement from 51.4% to 94.7% in user-specific scenarios, with an overall latency of 338 ms. The use of user-specific models is, however, not recommended in real-life contexts, where the need to record several sessions of movement repetitions to train the model before using it is excessively time-consuming. In this regard, the best compromise might be a calibration routine for user-specific fine-tuning of a pre-trained user-general model, as proposed in [59,60].

In [41], on the other hand, the performance of user-specific and user-general models are both documented, with the substantial classification accuracy increases reported in Table 3. However, their orientation correction method was tested only offline, leveraging a dataset of 612 users divided 50/50 between training and test sets, and the real-time data acquisition was simulated using a sliding window approach. However, simulating real-time acquisition with sliding windows may overlook important factors, such as wireless communication delays and computational complexity, which might also prevent a more in-depth analysis necessary for optimizing system performance with implementations at the edge. Therefore, the reported classification times of 16.97 ms and 71.69 ms should not be accounted for as response times alone and should not be used as the only measure to prove the suitability for a real-time application.

Another promising solution is the one proposed in [26], where the authors, using the Myo armband, proposed an ANN trained with different sEMG time-domain features for recognizing nine hand gestures. The training was carried out by integrating the sEMG data sensed with the armband worn with different angular orientations to enable the model to handle electrode-shifted signals. Their offline validation analysis on ten subjects brought promising results, achieving an average classification accuracy of 99.3%. They also demonstrated, although not completing the device integration, the possibility of embedding their method onto a Raspberry Pi 3 B+ platform with good performance, considering the latency for feature extraction and classification tasks.

In contrast to the three previous works, which used the Myo armband, in [43] a novel custom wearable device was used for sEMG acquisitions with a total of 64 input channels. Here, again, the usage was not tested in an online setting and the reported performance was user-specific, with a good average accuracy increase from 52.92% to 78.30% across 12 healthy participants.

In this work, we embedded the proposed shift/reversal correction method in our custom armband and tested it online with 25 users not involved in the training phase. Starting from an accuracy of 83.8% obtained from the preliminary analysis described in Section 3, using our correction method, we achieved a classification accuracy of 93.4% during in vivo online testing. The achieved value is comparable with the best ones obtained from the comparative works, and the presented calibration also enables the handling of an additional degree of freedom in wearing the armband. Indeed, instead of only addressing the shift of the device (DOF_1_), we also took into account the possibility of reversing the armband back-to-front (DOF_3_). Last, with an overall latency of just 181.75 ms, including only 0.408 ms for input data correction, our solution proves significant potential for real-time applications.

## 7. Conclusions and Final Remarks

In this work, we proposed an algorithm to estimate the orientation of sEMG armbands around the forearm. These devices are commonly used for implementing HMI systems based on the hand gesture classification approach, and they usually exploit the benefits of machine learning models to account for people’s diversity. However, these systems significantly decrease performance when users wear the device multiple times. One primary reason is the electrode-to-muscle shift, which can occur involuntarily during re-wearing and distort the causality between the detected muscular signals and the hand gestures to be recognized.

In order to solve this issue, we developed a two-phase algorithm to first estimate the orientation of the armband and then correct the muscular-sensed data. The first phase, the calibration process, requires the user to perform two reference movements for a short time (i.e., less than 1 min) to compute both the angular shift of the armband around the forearm and its possible reversal wearing condition. The second phase, which takes place when the armband is working to recognize hand gestures, uses the calibration data to correct the misplaced muscle information and then passes it to the machine learning model responsible for the recognition. The proposed approach has been designed to work with any sEMG sensing armband and does not require any machine learning re-training process or new data acquisition campaigns. Indeed, it could be applied to publicly available databases and easily implemented into existing solutions. The main code routines needed to estimate and correct the armband orientation consist of simple mathematical computations (e.g., signal correlation between small-size data arrays), and they can be embedded into resource-limited hardware devices.

We demonstrated the efficacy of this algorithm by implementing it on our custom seven-channel sEMG-based armband [28], which embeds an ANN trained to recognize nine hand gestures. Our experimental validation process involved 25 subjects, who repeated the test session two times while arbitrarily wearing the armband at the beginning of each one. As the result of these tests, we achieved an average accuracy of 93.36%, comparable if not slightly better than that obtained in our previous work [28], i.e., 91.93%, where, however, unlike this work, the reference wearing position was respected. Moreover, we also proved the low-power and low-latency feature of the proposed algorithm: we measured an increment of about 500 μW and of 0.408 ms for power consumption and application latency, respectively, during online classification w.r.t our previous study [28]. These results confirm the general lightness of this orientation correction algorithm, which does not impact pre-existing routines and, in our case, still satisfies the power and latency requirements of a typical hand gesture recognition real-time HMI system.

All the above considered, we believe that the proposed algorithm can be helpful in several works to solve the common problem of the electrode shift. Although it has to be conformed and optimized for any specific use case, the methods and applicability conditions presented here have been defined for a general scenario involving sEMG armbands, making this algorithm suitable for different solutions. Indeed, considering the overall lightness and simplicity of this approach, as demonstrated by the computational, efficiency, and timing aspects, combined with a minimal effort for a developer to include it in her/his own projects, e.g., new data campaigns or models retraining are not required, a wide range of applications can effectively adopt it. 

## Figures and Tables

**Table 1:** Used attributes from comparative databases.

Name	Subjects	Devices	Available Data
DB1	27 healthy	8 OttoBock	RMS
DB2	40 healthy	8 Delsys	sEMG
DB3	8 people with amputations	8 Delsys	sEMG
DB4	10 healthy	8 Cometa	sEMG
DB5	10 healthy	1 Myo armband (8 channels)	sEMG
Ours	20 healthy	1 custom armband (7 channels)	ATC

**Table 2:** Reversal correction: settings and assessment.

Name	Condition A	Condition B	D_ch_	MAE_fw_	MAE_bw_	flag_bw_
DB1	WE	HG	3	41.9	30.1	1
DB2	WE	UD	1	26.4	13.1	1
DB3	HG	WF	2	15.3	32.0	0
DB4	WE	UD	1	16.4	23.1	0
DB5	WE	HG	2	19.0	38.9	0
Ours	WE	UD	1	7.6	2.1	1

**Table 3:** SoA comparison of orientation-robust sEMG-based armbands.

Work	Year	# Channels	# Gestures	Classifier Type	Device	Embedded Algorithm	DOFs Handled	Online Latency	# Tested Subjects	Accuracy Increase
[16]	2020	8	6	ANN	Myo armband	✗	1	338 ms	10	51.4% → 94.7% ^⋆^
[41]	2020	8	6	SVM ^1^	Myo armband	✗	1	Offline	306	44.5% → 81.2% ^⋆^ 39.8% → 94.9% ^⋄^
[26]	2020	8	9	ANN	Myo armband	✓	1	Offline	10	N.A. → 99.3% ^⋄^
[43]	2023	64	6	CNN ^2^	Custom	✗	1	Offline	12	52.9% → 78.3% ^⋆^
This	2024	7	9	ANN	Custom	✓	1 & 3	182 ms	25	83.8% → 93.4% ^⋄^

^1^ Support Vector Machine, ^2^ Convolutional Neural Network. ^⋆^ User-specific, ^⋄^ User-general.

## Data Availability

The Ninapro public datasets are available at https://ninapro.hevs.ch/, accessed on 7 February 2025. The ATC dataset used in this study is available upon request from the corresponding author. The ATC dataset is not publicly available because of future studies involving it.
